# Modulation of Biofilm-Formation in *Salmonella enterica* Serovar Typhimurium by the Periplasmic DsbA/DsbB Oxidoreductase System Requires the GGDEF-EAL Domain Protein STM3615

**DOI:** 10.1371/journal.pone.0106095

**Published:** 2014-08-25

**Authors:** Naeem Anwar, Syed Fazle Rouf, Ute Römling, Mikael Rhen

**Affiliations:** 1 Department of Microbiology, Tumor and Cell Biology, Karolinska Institutet, Stockholm, Sweden; 2 Department of Biology, Syed Babar Ali School of Science and Engineering, Lahore University of Management Sciences, Lahore, Pakistan; University of Osnabrueck, Germany

## Abstract

In *Salmonella enterica* serovar Typhimurium (*S*. Typhimurium), biofilm-formation is controlled by the cytoplasmic intracellular small-molecular second messenger cyclic 3′, 5′-di- guanosine monophosphate (c-di-GMP) through the activities of GGDEF and EAL domain proteins. Here we describe that deleting either *dsbA* or *dsbB*, respectively encoding a periplasmic protein disulfide oxidase and a cytoplasmic membrane disulfide oxidoreductase, resulted in increased biofilm-formation on solid medium. This increased biofilm-formation, defined as a *r*ed, *d*ry *a*nd *r*ough (*rdar*) colony morphotype, paralleled with enhanced expression of the biofilm master regulator CsgD and the biofilm-associated fimbrial subunit CsgA. Deleting *csgD* in either *dsb* mutant abrogated the enhanced biofilm-formation. Likewise, overexpression of the c-di-GMP phosphodiesterase YhjH, or mutationally inactivating the CsgD activator EAL-domain protein YdiV, reduced biofilm-formation in either of the *dsb* mutants. Intriguingly, deleting the GGDEF-EAL domain protein gene *STM3615 (yhjK)*, previously not connected to *rdar* morphotype development, also abrogated the escalated *rdar* morphotype formation in *dsb* mutant backgrounds. Enhanced biofilm-formation in *dsb* mutants was furthermore annulled by exposure to the protein disulfide catalyst copper chloride. When analyzed for the effect of exogenous reducing stress on biofilm-formation, both *dsb* mutants initially showed an escalated *rdar* morphotype development that later dissolved to reveal a smooth mucoid colony morphotype. From these results we conclude that biofilm-development in *S.* Typhimurium is affected by periplasmic protein disulphide bond status through CsgD, and discuss the involvement of selected GGDEF/EAL domain protein(s) as signaling mediators.

## Introduction

Infections with *Salmonella enterica* (*S. enterica*) represent a major health problem and a significant burden on food industry [1]. In the European Union alone, infections caused by *S. enterica* serovar Typhimurium (*S.* Typhimurium) stand as the second most prevalent cause of food-born acute gastroenteritis [2]. In human, infections related to non-typhoidal serovariants, such as *S.* Typhimurium, are usually seen as an acute self-healing infection [3]. In contrast, typhoid fever, caused by *S. enterica* serovar Typhi (*S*. Typhi), represents a severe and potentially lethal systemic infection [3].

Murine salmonellosis as caused by *S*. Typhimurium [4], as well as cell culture infections using the pathogen, have been used as models for dissecting the details of invasive systemic salmonellosis. In this, several virulence factors have been identified as instrumental in steering the acute systemic infection [3]. A hallmark of salmonellosis, whether non-typhoidal or typhoidal, is the establishment of carrier states [5], [6]. In fact, 3 to 5% of the convalescent typhoid fever cases are converted to asymptomatic carriers [7], [8] while animals, such as reptiles, may harbor and shed non-typhoidal *Salmonellae* for prolonged periods of time [9]. Such carrier states are likely responsible for transmission and continuous outbreaks of salmonellosis [5], [7]. Details governing this very important step of salmonellosis have remained much less explored. However many virulence factors identified to act in the acute phase of the infection also contribute to persistency, including factors mediating tolerance to oxidative stress [10]. In addition, the formation of a so-called biofilm on cholesterol-rich gallstones is believed to promote persistent carriage both in murine infection models, as well as in man [11].

Bacterial biofilms are complex communities consisting of microorganisms embedded in a self-produced extracellular matrix. In this matrix, microbes grow on either biotic or abiotic surfaces, attaching to the surface and each other, conferring resistance to both immunity-related as well as pharmaceutical antimicrobials [12].

Apart from being a probe for microbial pathogenesis, *S*. Typhimurium is a well-defined model organism for detailing events in bacterial biofilm-formation. For *S*. Typhimurium, biofilm-formation is characterized by a ***r***ed ***d***ry ***a***nd ***r***ough (*rdar*) morphotype when grown on low-osmomolarity nutrient agar plates supplemented with the diazo dye Congo red [12]. Formation of the *rdar* morphotype much relies on the production of the extracellular matrix components cellulose and so-called curli fimbriae consisting of the CsgA as the major protein subunit [13].

The transition into an *rdar* morphotype relies on the biofilm master gene regulator protein CsgD. CsgD activates the *csgBAC* operon with accompanied increased production of the curli fimbrial CsgA and CsgB subunits [14], [15]. Further, CsgD indirectly increases cellulose production by activating *adrA* that codes for a di-guanylate cyclase [15]. The small molecule cyclic di-guanosine monophosphate (c-di-GMP) generated by AdrA is a ubiquitous secondary messenger found in almost all bacterial species [16]–[18]. The AdrA-mediated increase in c-di-GMP enhances expression of the cellulose synthetase BcsA, which in turn increases cellulose production [14], [19].

The cellular levels of c-di-GMP are maintained by GGDEF and EAL/HD-GYP domain proteins, which act as diguanylate cyclases and phosphodiesterases respectively [20]–[23]. Contrary to sessility, motility is inhibited by increased levels of c-di-GMP [23]. Hence, increased cellular levels of c-di-GMP promote a sessile growth of bacteria [20], [21]. Activation of motility is also regarded as initiating egression from biofilm-formation to allow for further colonization of new habitats [24].

Interestingly, in a number of bacteria a substantial number of genes that are affected during switches between planktonic and sessile growth are connected to oxidative stress tolerance [25]–[27]. Moreover, Wang and colleagues reported that oxidative stress up-regulate biofilm related genes in *S. enterica* [28]. Relevant for the topic, oxidative stress is also an important arm of innate defense against salmonellosis in both men and mice, and many bacterial oxidoreductases strongly contribute to virulence in cell culture and murine infections models [29]–[33]. Moreover, another innate radical-based defense, nitric oxide (NO), inhibits biofilm-formation in *Pseudomonas aeruginosa* [34], and in *Shewanella oneidensis* through interference with c-di-GMP signaling [35].

There are three well-defined oxidoreductase systems in *Escherichia coli* that maintain proper protein disulphide bond formation and that cope with oxidative stress; the gluthione/glutaredoxin system, the thioredoxin system and the ***d***isulfide ***b***ond ***s***ystem (Dsb), the executing enzymes of which all belong to the thioredoxin superfamily characterized by a Cys-X-X-Cys catalytic motif. The former two systems are operational in the cytoplasm while later maintains proper disulfide bond status of periplasm, yet receiving its reducing equivalents either from the glutaredoxin or thioredoxin pathways [36]–[39]. Based on genome sequence annotations, the very same enzymes are also present in *S*. Typhimurium [40]–[43].

Biofilm-formation also appears connected to redox enzymes [44]–[48]. The periplasmic superoxide dismutase (SOD) is essential for biofilm-formation in *E. coli* and *Listeria monocytogenes* [44], [48], while in *E. coli* the periplasmic disulphide oxidase DsbA is essential for biofilm-formation during growth in static broth growth [45]. Here we have dissected the contribution of forth-mentioned redox systems to the biofilm-formation in *S*. Typhimurium and point to a mechanistic connection between the Dsb system, c-di-GMP and the biofilm master regulator CsgD.

## Results

### Oxidoreductases and *rdar* morphotype development in *S*. Typhimurium

To map the role of oxidoreductases of the thioredoxin superfamily in *S.* Typhimurium in biofilm-formation, we started by collecting and constructing individual *ΔdsbA, ΔdsbB, ΔdsbC, ΔdsbD, ΔdsbI, ΔdsbL, ΔgshA*, *ΔgrxB, ΔtrxA* and *ΔtrxB* mutants in the *S*. Typhimurium 14028 background, thus generating a set of mutants formally defective in any of the three oxidoreductase systems introduced above. In contrast to laboratory *E. coli* strains, *S*. Typhimurium codes for the additional thioredoxin-like ScsABCD proteins [49], [50]. Finally, several strains of *S.* Typhimurium carry a “virulence-associated” plasmid (pSLT) that codes for the DsbA paralogue SrgA [40]. Therefore, we included a *ΔscsABCD* mutant and an isogenic *S*. Typhimurium strain pair carrying or lacking pSLT into the strain panel.

**Figure 1:**
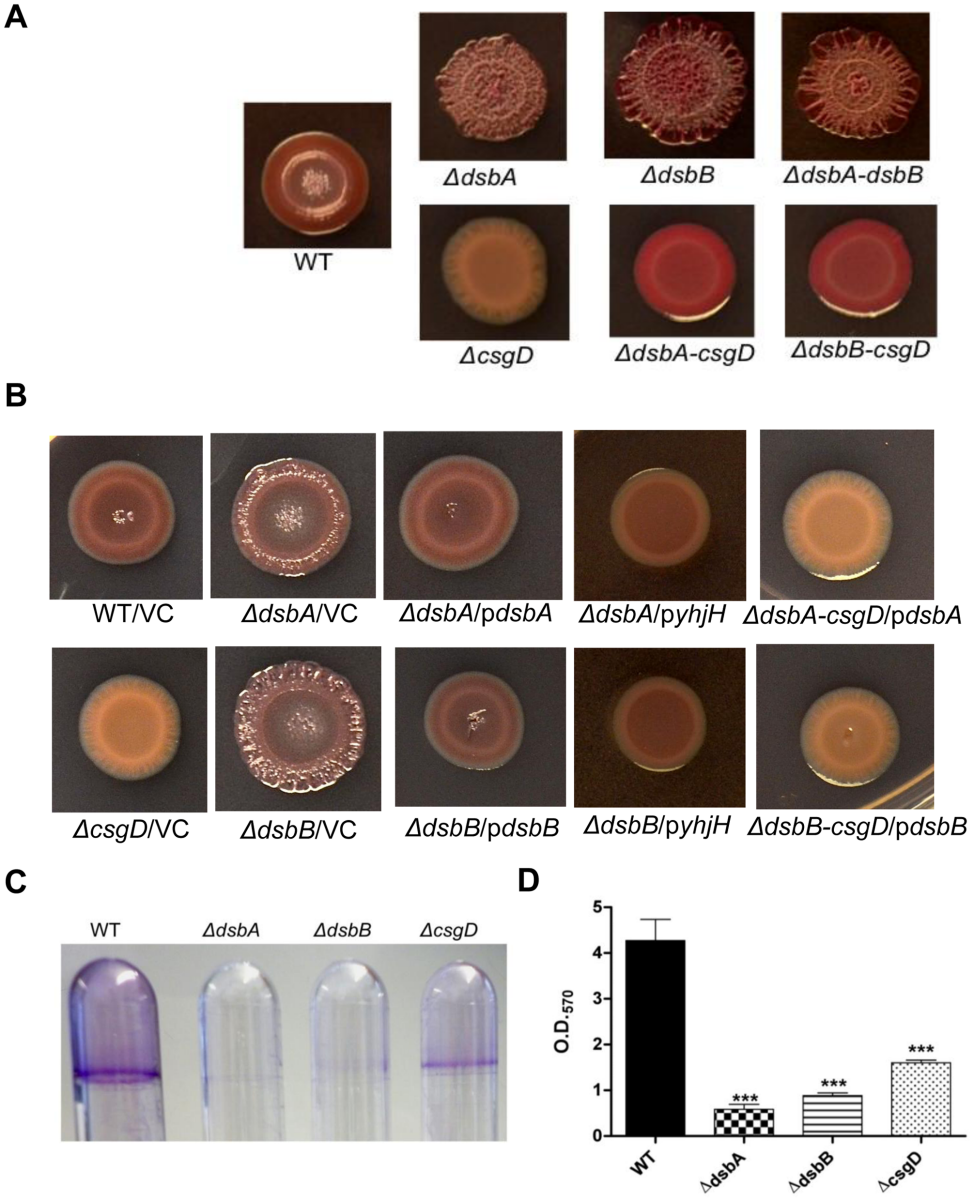
Biofilm-formation on solid media and liquid media. The formation of *rdar* morphotype in wild type, single and double *dsb* mutants grown for 48 hours on Congo red plates at 28°C is illustrated in panel (**A**). This escalated rdar morphotype development can be reverted to wild type level by *trans*-complementation with corresponding cloned *dsb* genes, or by introducing a cloned *yhjH* gene or depleting *csgD* (**B**). VC indicates the vector control, pBAD30. **C**) Crystal violet staining of biofilm adherent to polystyrene tubes as an indicator of biofilm-formation at liquid-air interface. The *ΔdsbA* and *ΔdsbB* mutants fail to make a pellicle at air-liquid interface in static LB without salt culture at 28°C 24 hours post inoculation. **D**) Quantification of adherent biofilm measured as retained Crystal violet in biofilm. Error bars indicate SEM. ***  = p≤0.001.

Next, each mutant was compared with the wild type for *rdar* morphotype development on congo red (CR) agar plates. In this, the *ΔdsbA* and *ΔdsbB* mutants revealed a marked escalation in *rdar* morphotype development (Fig. 1A and S1). While we noted minor alterations in *rdar* morphotype development for some of the additional mutants, none of these deviations were comparable in degree with that of the *ΔdsbA* and *ΔdsbB* mutants (Fig. S1). Also, the strain pair proficient or deficient in pSLT showed an identical *rdar* morphotype development (Fig. S1). Hence, we focused on *dsbA* and *dsbB* in further study.

The *rdar* morphotype development in a *ΔdsbA*-*dsbB* double mutant behaved as either of the single *dsbA* or *dsbB* deletion mutants (Fig. 1A), implying that the *dsbA* and *dsbB* acted through a convergent rather than through divergent pathways in suppressing *rdar* morphotype development. To exclude any genetic downstream effect of the mutations introduced, we showed restoration of wild type *rdar* formation by providing a cloned *dsbA* or *dsbB* gene into the respective single mutant (Fig. 1B).

In parallel, somewhat surprisingly, we noted that both the *ΔdsbA* and *ΔdsbB* mutant failed to create pellicles in static salt-less LB broth culture at the air-liquid interface another type of biofilm mediated by the *rdar* morphotype (Fig. 1C and 1D) [51]. Combined, these observations implied that biofilm-formation as assayed by *rdar* morphotype or by pellicle formation differentially relied on the DsbA/DsbB redox-shuffling system.

### DsbA and DsbB affect expression of CsgA and CsgD

The *rdar* morphotype of *S.* Typhimurium mainly develops through the expression of surface-located curli fimbriae and cellulose in the extracellular matrix [12]. CsgA (AgfA) is the major structural subunit of these fimbriae [52], [53], the expression of which is positively regulated by CsgD, the major transcriptional activator of biofilm-formation in *E. coli* and *S. enterica* [54]. Therefore, we extended our study to determine the effect of *ΔdsbA* and *ΔdsbB* mutations on CsgA and CsgD expression.

**Figure 2:**
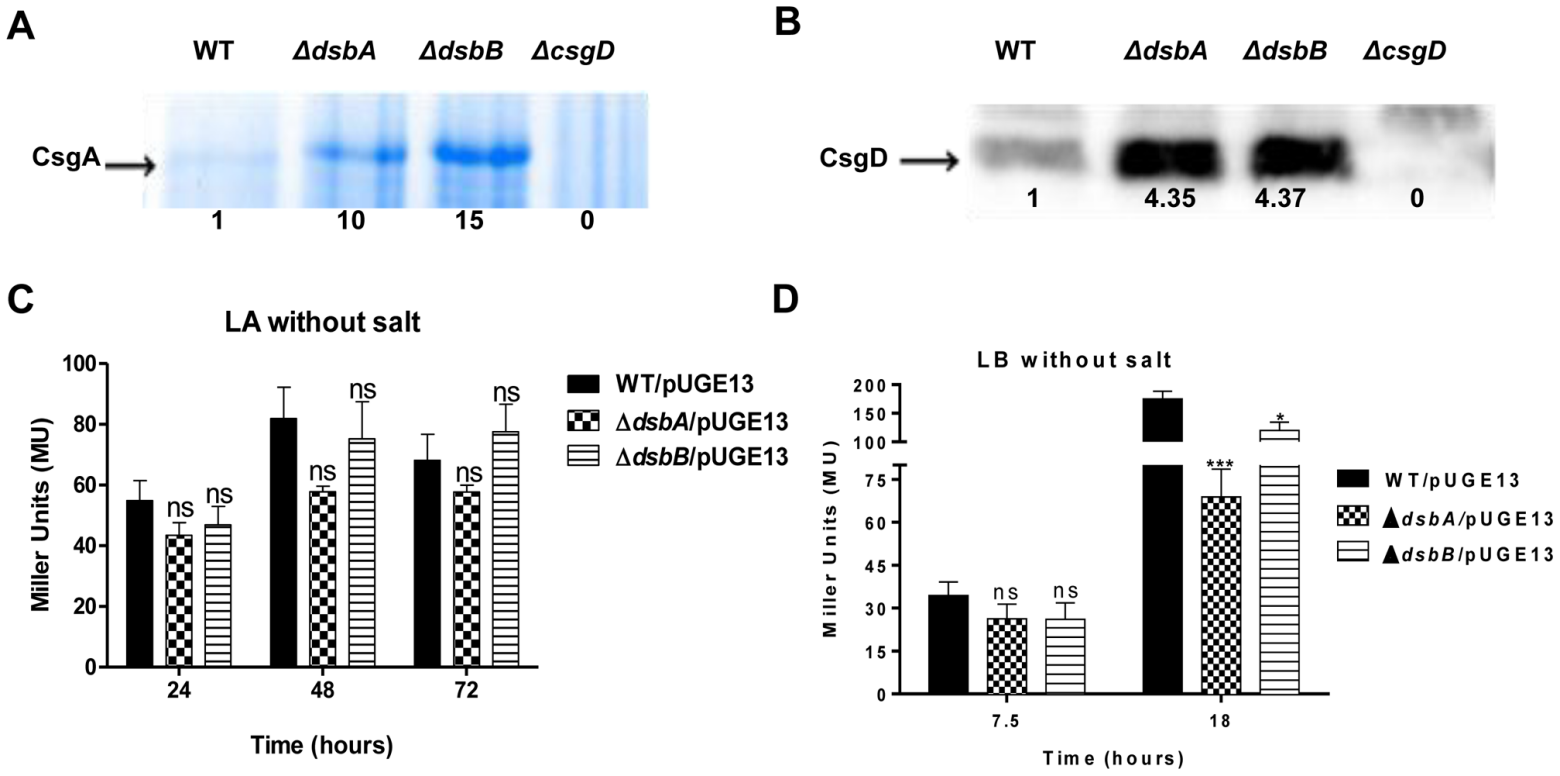
Effect of *ΔdsbA* and *ΔdsbB* mutations on the expression of CsgA and CsgD. **A**) Coomassie blue stained SDS-PAGE gel revealing increased CsgA production in *ΔdsbA* and *ΔdsbB* mutants when grown on LA without salt at 28°C at 24 hours of incubation. **B**) Immunoblot for CsgD shows increased level of CsgD for *ΔdsbA* and *ΔdsbB* mutants from the same bacterial cultures. Numbers underneath the lanes in **A**) and **B**) specifies the relative band intensities. The *csgD-lacZ* promoter fusion activity at 18 hours post inoculation from salt-less Luria Agar cultures (**C**) or from salt-less Luria broth cultures (**D**) grown at 28°C. Error bars indicate SEM. ***  = p≤0.001; **  = p≤0.01; ns  =  not significant compared to respective wild type.

CsgA was isolated from cultures grown on Luria agar (LA) plates without salt for two days at 28°C until a rugous morphotype development was evident. CsgA was then extracted and depolymerized with the use of formic acid before the preparations were run on SDS-PAGE gels to compare CsgA yields. In this analysis, the CsgA yields were around 10 times higher in the *ΔdsbA* mutant and around 15 times higher in the *ΔdsbB* mutant as compared to the isogenic wild type control (Fig. 2A). Thus, the increase in CsgA levels was in accordance with the *rdar* morhoptype of the mutants on CR plates (Fig. 1A). Similarly, the CsgD levels, as determined by immunoblot analysis from the cellular fraction of same cultures, were about 2–4 times higher in both the *ΔdsbA* and *ΔdsbB* mutants (Fig. 2B). However, we could not detect CsgA or CsgD from the static liquid cultures, whether using bacterial extracts from wild type or *dsb* mutants (data not shown).

To test whether *dsbA* or *dsbB* affected CsgD levels at the transcriptional or post-transcriptional level, we measured the promoter activity of *csgD* by using a plasmid-carried *csgD-lacZ* transcriptional promoter fusion [55]. In this analysis, we could not detect any substantial differences in *csgD* promoter activity from salt free LA plate cultures at any time point for either mutant (Fig. 2C). These results suggest that on plates, *ΔdsbA* and *ΔdsbB* mutants affect CsgD expression at a post-transcriptional level. Surprisingly though, in broth cultures under shaking conditions, we noted a decrease in *csgD-lacZ* activity for either *dsb* mutant that is consistent with the decreased pellicle formation of the *dsb* mutants (Fig. 2D).

### CsgD- and c-di-GMP-dependency of *ΔdsbA* and *ΔdsbB* mutant *rdar* morphotypes

**Figure 3:**
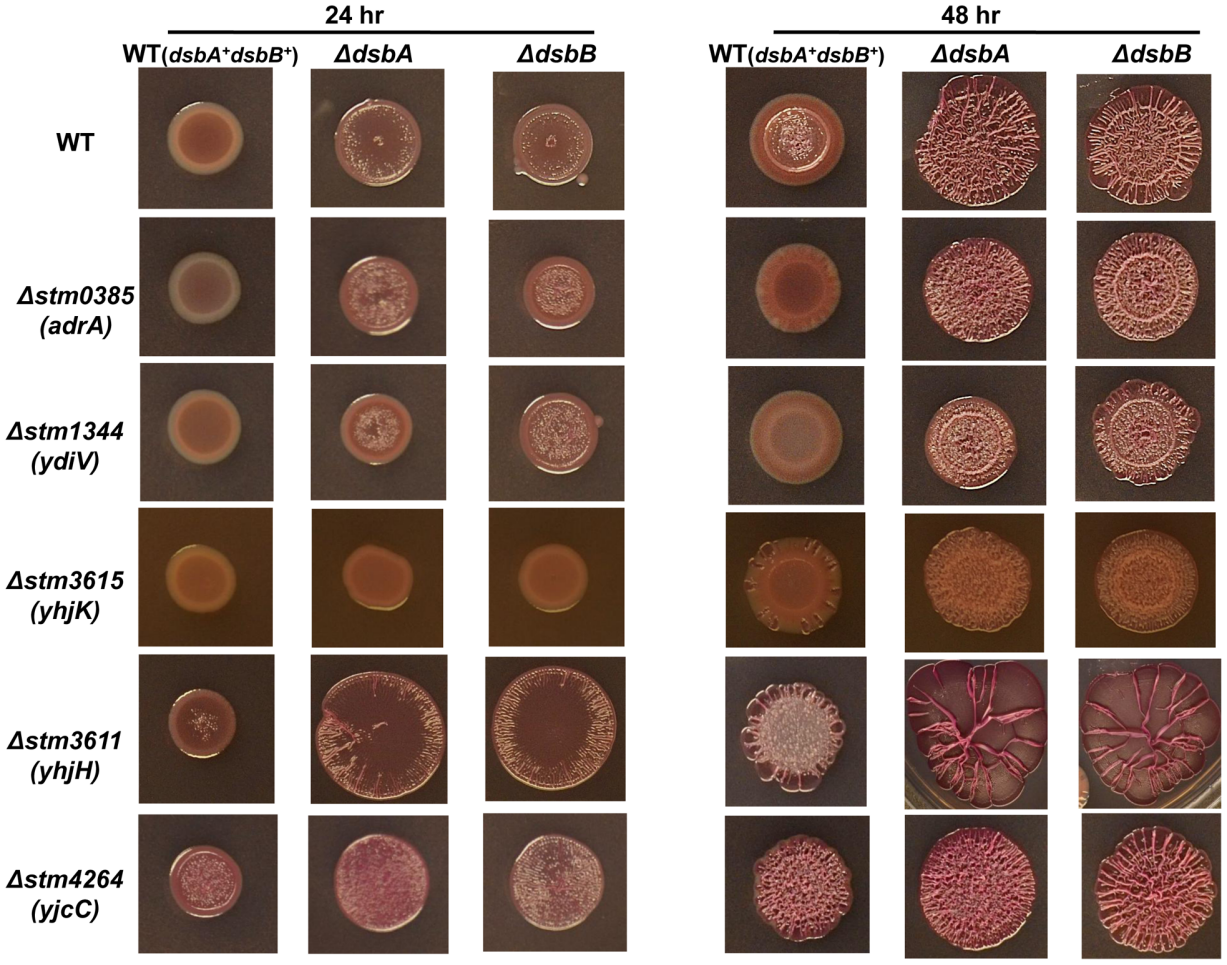
Effect of GGDEF/EAL protein gene mutations on *dsb* associated biofilm-formation. The development of *rdar* morphotype in wild type, single and double mutants on Congo red plates at 28°C after 24 and 48 hours of incubation.

As stated above, *rdar* morphotype development is stimulated by c-di-GMP [54], while GGDEF and EAL domain proteins in turn balance the levels of c-di-GMP [54], [55]. Moreover, recent reports show that selected GGDEF/EAL domains proteins in *E. coli* activate the promoter of the *csgBAC* operon independent of CsgD [58], [59]. As DsbA and DsbB appeared to affect the levels of CsgD (Fig. 2B), we first asked whether the effect of the *ΔdsbA* and *ΔdsbB* mutations on *rdar* morphotype development required CsgD. Hence, to probe for a role of CsgD in the escalated *rdar* morphotype development in the *dsb* mutants, we deleted *dsbA* or *dsbB* in a *ΔcsgD* mutant background. Both double mutants expressed a red color, but failed in *rdar* morphotype development on the Congo red (CR) plates (Fig. 1A). This characteristic Congo red binding of *ΔdsbA-csgD* and *ΔdsbB-csgD* double mutants most likely implicates residual production of the extracellular matrix component cellulose (Fig. 1A and 3).

Next, we determined whether the enhanced *rdar* morphotype formation of the *ΔdsbA* or *ΔdsbB* mutants was released from c-di-GMP mediated control. To address this, we used an indirect approach. The *ΔdsbA* and *ΔdsbB* mutants were transformed with a cloned plasmid containing the phosphodiesterase gene *yhjH*, under an arabinose inducible promoter. When the *ΔdsbA* and *ΔdsbB* mutants were grown under conditions inducing *yhjH*, formally depleting the bacterial cells of c-di-GMP, the increased *rdar* morphotype development of the two *dsb* mutants were decreased while no such effect was observed using the vector control (Fig. 1B). Thus, the escalated *rdar* morphotype development of *ΔdsbA* or *ΔdsbB* mutants was not due to a plain disconnection from CsgD or c-di-GMP-mediated control.

### GGDEF/EAL domain proteins and *rdar* morphotype development

To date 20 GGDEF/EAL domain proteins have been identified in *S*. Typhimurium [60]. As selected housekeeping functions in *S*. Typhimurium affect *rdar* morphotype development through selected GGDEF/EAL domain proteins such as CsgD, and as the elevated *rdar* morphotype development could be down-regulated by the c-di-GMP phosphodiesterases YhjH in both the *ΔdsbA* or *ΔdsbB* mutant, we investigated to identify GGDEF/EAL domain protein(s) are required for the up-regulated *rdar* morphotype in *ΔdsbA*/*B* mutant. To this end, we created double mutants all of the twenty GGDEF/EAL domain protein genes in the *ΔdsbA* or *ΔdsbB* mutant backgrounds. Subsequently, we compared *rdar* morhotype development in single and all combinations of double mutants (Table 1, Fig. 3).

**Table 1:** Effect of GGDEF/EAL proteins on *dsb* associated biofilm-formation.

Mutation	Background					
	WT(*dsbA^+^dsbB^+^*)		*ΔdsbA*		*ΔdsbB*	
	24hr	48hr	24hr	48hr	24hr	48hr
WT	+/−	+	+	2+	+	2+
*Δstm1142 (csgD)*	−	−	−	−	−	−
*Δstm0385 (adrA)*	−	−	+	2+	+	2+
*Δstm1283 (yeaJ)*	−	−	+	2+	+	2+
*Δstm1987 (yedQ)*	−	−	+	2+	+	2+
*Δstm2672 (yfiN)*	−	−	+	2+	+	2+
*Δstm4551*	−	−	+	2+	+	2+
*Δstm2123 (yegE)*	−	−	+	2+	+	2+
*Δstm3388*	−	−	+	2+	+	2+
*Δstm1697*	−	+	+	2+	+	2+
*Δstm3375 (csrD)*	+/−	+	+	2+	+	2+
*Δstm1344 (ydiV)*	−	−	+	1.5+	+	1.5+
*Δstm3615 (yhjK)*	+/−	+	+/−	1.5+	+/−	1.5+
*Δstm0343*	+/−	+	+	2+	+	2+
*Δstm0468 (ylaB)*	+/−	+	+	2+	+	2+
*Δstm1827*	+	2+	2+	3+	2+	3+
*Δstm2215 (rtn)*	+	2+	2+	3+	2+	3+
*Δstm4264 (yjcC)*	+	2+	2+	3+	2+	3+
*Δstm3611 (yhjH)*	+	2+	2+	4+	2+	4+
*Δstm1703 (yciR)*	2+	3+	2+	4+	2+	4+
*Δstm2410 (yfeA)*	+	2+	+	2+	+	2+
*Δstm2503 (yfgF)*	+	2+	+	2+	+	2+
*Δstm1344–3615*	+/−	+	+/−	+	+/−	+

−/+/2+/3+ etc. represents the degree of the biofilm-formation compared to the WT.

The GGDEF/EAL domain protein *STM1703* (*yciR*) and GGDEF domain proteins *STM3611* (*yhjH*) and *STM4264* (*yjcC*) each inhibit expression of CsgD and hence *rdar* morphotype development [57]. Accordingly, as also noted here, plain *yciR, yhjH* and *yjcC* mutants revealed an escalated *rdar* morphotype development. In parallel, we noted a further escalation in *rdar* morphotype development for corresponding *ΔdsbA* and *ΔdsbB* mutants in the *ΔyciR* or *ΔyhjH* mutant backgrounds at 48 hours post inoculation, while *ΔdsbA-yjcC* and *ΔdsbB-yjcC* double mutants much behaved as single *dsb* mutants (Table 1, Fig. 3).

In accordance with prevailing literature, single mutants lacking *STM1344* (*ydiV*), *STM2123* (*yegE*), *STM2672* (*yfiN*), *STM3388* or *STM4551* expressed a strongly reduced *rdar* morphotype development (Table 1, Fig. 3). Still, when the *ΔdsbA* or *ΔdsbB* deletions were introduced into the *ΔyegE* or *ΔyfiN* mutant backgrounds we did not note any substantially reduced *rdar* morphotype development compared to single *ΔdsbA* or *ΔdsbB* mutants (Table 1). In contrast, the *ΔdsbA-ydiV* and *ΔdsbB-ydiV* double mutants revealed a delayed *rdar* morphotype development in comparison to plain *ΔdsbA* or *ΔdsbB* mutants (Table 1, Fig. 3), notably at 24 hours post inoculation. Intriguingly, while not affecting *rdar* morphotype development in a wild type background, deleting *STM3615* (*yhjK*) in either the *ΔdsbA* or *ΔdsbB* mutant resulted in a delayed *rdar* morphotype development (Table 1, Fig. 3).

**Figure 4:**
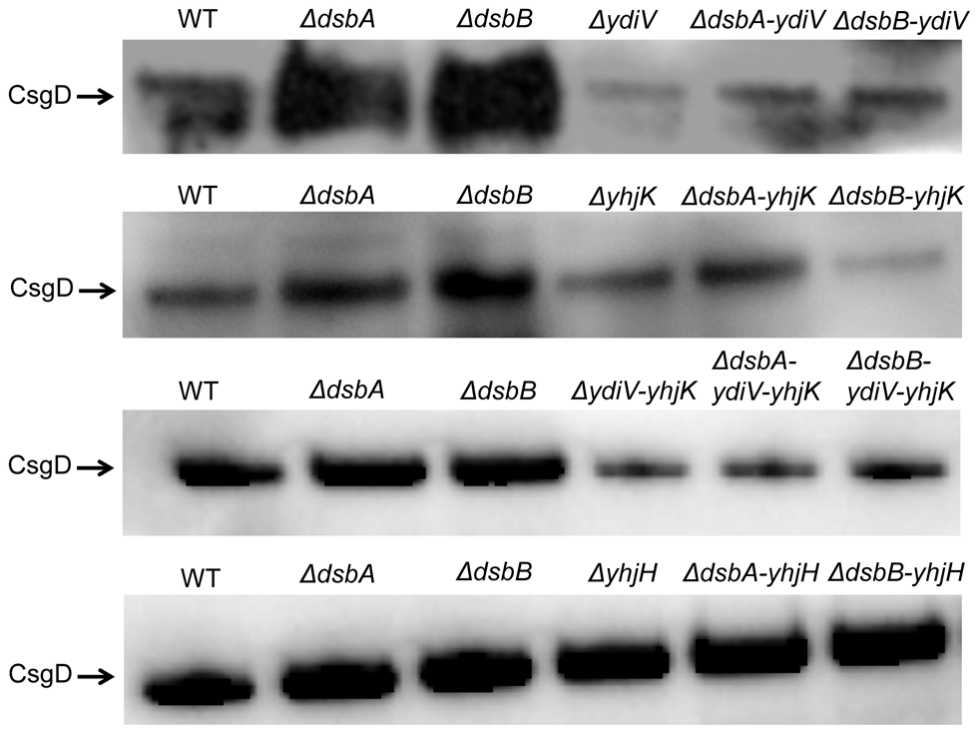
Effect of *ydiV*, *yhjK* and *yhjH* mutations on *dsb* associated biofilm-formation. Immunoblot for CsgD from overnight salt-less Luria Agar cultures shows altered level of CsgD expression for *ΔdsbA* and *ΔdsbB* mutants by introducing mutations in *ydiV*, *yhjK, ydiV-yhjK*, and *yhjH* genes respectively.

When assaying for CsgD expression in the *ΔydiV* and *ΔyhjK* mutants, we noted decreased CsgD levels in the *ΔydiV*, *ΔdsbA-ydiV* and *ΔdsbB-ydiV* mutants as compared to the wild type and single *dsb* mutants (Fig. 4). In contrast, a plain *ΔyhjK* mutant did not reveal any obvious alteration in CsgD expression, while the double mutants particularly *ΔdsbB-yhjK* double mutant did reveal a decrease in CsgD levels. Furthermore, the CsgD expression and *rdar* morphotype in the *ΔydiV-yhjK and ΔydiV-yhjK-dsbA/B* mutants was also reduced to the respective wild type and single *dsb* mutants (Fig. 4, Fig S2). However, the *ΔyhjH* and *ΔyhjH-dsbA/B* mutants showed induced CsgD levels as compared to the wild type (Fig. 4). Thus, the CsgD levels closely correlated with *rdar* morphotype development of respective strains.

### Oxidative and reductive stresses disperse *rdar* morphotype development in *ΔdsbA* and *ΔdsbB* mutants

Bacterial biofilm-formation responds to oxidative and nitrosative stress [34], [35], [61], and relies on periplasmic oxidoreductase activity (Fig. 1A). Consequently, we tested whether the enhanced *rdar* morhotype development of *ΔdsbA* or *ΔdsbB* mutants could be affected by providing exogenous oxidative stress. Thus, we first supplemented the CR plates with the disulphide bond catalyst copper chloride (CuCl_2_) at non-lethal concentrations. Alternatively, sterile filter paper discs soaked with 1M CuCl_2_ were put at the very edge of bacterial inoculum streaked on CR plates. In this analysis, *S.* Typhimurium lost its ability to generate *rdar* morhotype upon CuCl_2_-exposure in a dose dependent manner, whether being proficient or deficient in *dsbA* or *dsbB* (Fig. S3).

**Figure 5:**
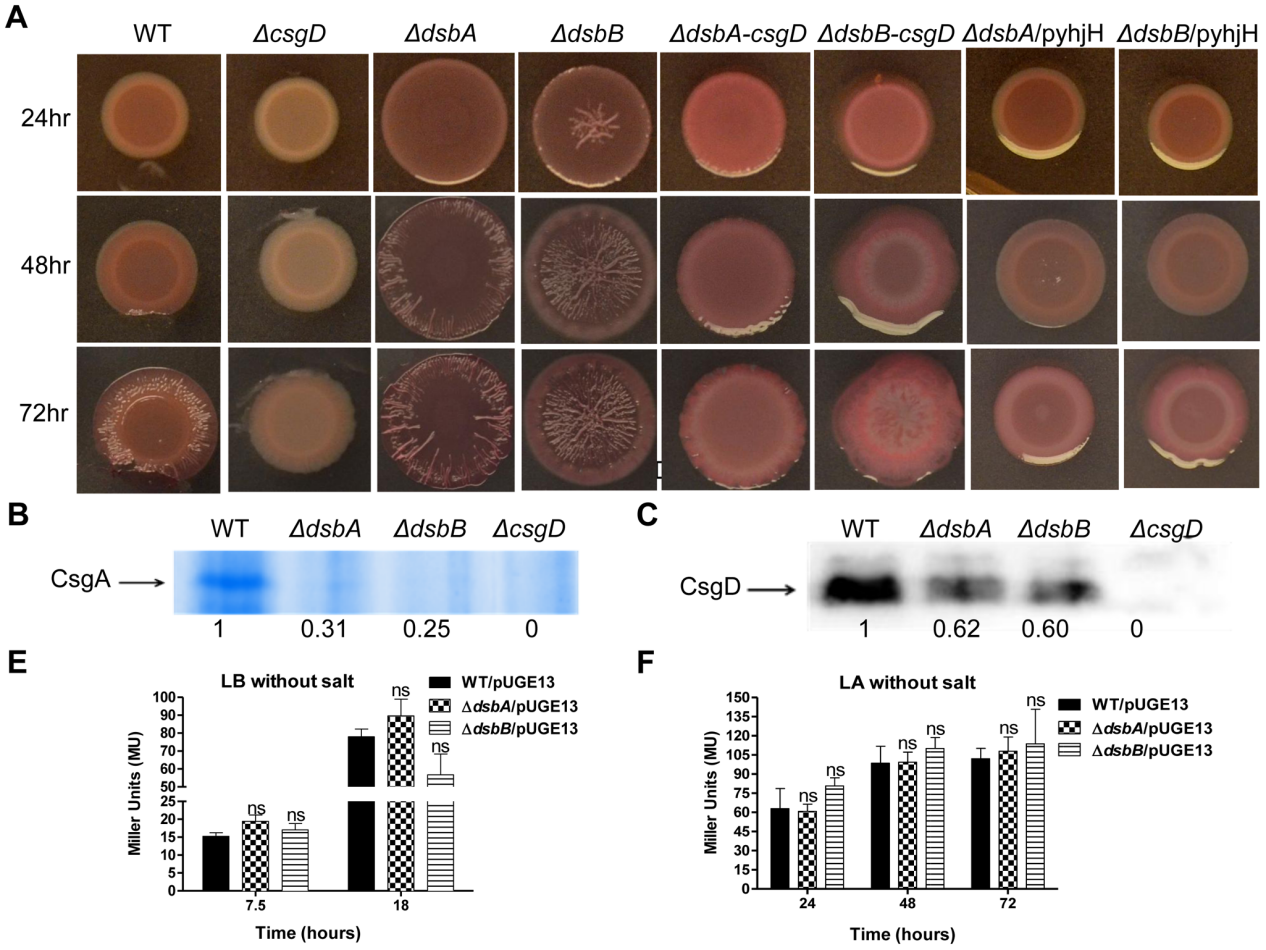
Effect of reducing stress on biofilm. **A**) Upon reductive stress (5 mM DTT) *ΔdsbA* and *ΔdsbB* mutants first shows an escalated *rdar* morpotype development, where after the appearance becomes smoother and mucoid. Overexpression of YhjH and deletion of *csgD* enhances slime production in *ΔdsbA* and *ΔdsbB* mutants under DTT reductive stress. Coomassie blue stained SDS-PAGE (**B**) and immunoblot (**C**) show decrease in the amounts of respectively CsgA and CsgD under 5 mM DTT stress from 18 hours at 28°C. The numbers underneath the lanes indicate relative amounts. **D**) and **E**) The *csgD-lacZ* promoter fusion activity in *ΔdsbA* and *ΔdsbB* mutants under 5 mM DTT stress in LB and LA cultures at 28°C. Error bars indicate SEM. ns  =  non-significant compared to respective wild type.

We next tested the reductant dithiothreitol (DTT) at a 5 mM final non-lethal concentration in the CR plates, after which we followed *rdar* morphotype development. When grown on such plates, the *dsb* mutants again showed enhanced *rdar* morphotype development at day one post inoculation, at which time the wild type strain still exhibited a rather smooth colony morphotype (Fig. 5A). At the second day post inoculation, both *dsb* mutants had lost their *rdar* morphotype, and converted into a highly mucoid morphotype three days post inoculation (Fig. 5A).

Complementation of either *ΔdsbA* or *ΔdsbB* mutant, with respective gene on a recombinant plasmid, under reductive stress, resulted in enhanced *rdar* morphotype development resembling the wild-type pattern (data not shown). Interestingly, the mucoid morphotype was enhanced in both the *ΔdsbA* and *ΔdsbB* mutant upon by knocking out *csgD* or upon expressing YhjH from the recombinant plasmid after 48 hours (Fig. 5A).

Accompanying the altered *rdar* morphotype development, the levels of CsgA and CsgD were reduced in *ΔdsbA* and *ΔdsbB* mutants as compared to the wild type strain under DTT stress (Fig. 5B and 5C). Still, we could not detect any effect of DTT stress on the CsgD promoter activity (Fig. 5D and 5E).

## Discussion

Several lines of evidence link redox stress with formation on bacterial biofilms [44]–[46], [61]. Here we demonstrate a role of the periplasmic Dsb oxidoreductase system in biofilm generation of *S*. Typhimurium. More precisely, we present that DsbA and DsbB negatively regulate biofilm-formation on solid growth medium but were required for the pellicle formation at the air-liquid interface in static broth, and that DsbA and DsbB prevent biofilm dispersion under reductive stress. We further indicate that *ΔdsbA* and *ΔdsbB* mutations affect expression of the major biofilm-activator protein CsgD and concomitantly expression of the biofilm extracellular matrix component CsgA.

Our results corroborate previous works in the sense that DsbA associates with biofilm-formation in *E*. *coli* O157 and *Pseudomonas putida*. For *E. coli* this dependence on DsbA materialized for biofilm-formation at the air liquid interface [45]. On the other hand, a *dsbA* mutant of *P. putida* expressed enhanced biofilm-formation in liquid medium [46]. Thus, the role DsbA for biofilm-formation of *E. coli* and *S.* Typhimurium in liquid media appears to contrast the requirement of DsbA for biofilm-formation in *P. putida* under similar conditions.

DsbA functions as a periplasmic disulphide bond catalyst, itself being reduced in the process. In *E. coli*, DsbA is returned into its oxidized state through DsbB [62]. As a pair, DsbA and DsbB are accounted responsible for no less than 40% of the *E. coli* envelope protein disulfide bond formation [63]. To the best of our knowledge, we could not find any report about the role of DsbB protein in biofilm-production. In our study, mutants deficient in either DsbA or DsbB exhibited an equal loss of biofilm-formation in broth, and in a comparable enhanced *rdar* morphotype development on plates that associated with an increased expression of CsgA and CsgD. As Δ*dsbA-csgD* and Δ*dsbB-csgD* double mutants failed to express any *rdar* morphotype, the increased CsgD expression in plain *dsb* mutants might well explain their enhanced *rdar* morphotype.

DsbA is not absolutely restrained in its substrate specificity. In fact, the non-specific disulphide catalyst CuCl_2_ can in part replace DsbA [64]. Therefore, we continued by testing the effect of CuCl_2_ on *rdar* morphotype development. CuCl_2_ totally abolished biofilm-development in *S.* Typhimurium whether the bacteria were proficient in *dsbA* or *dsbB*, or not. This observation adjusts well with an idea stating that a high periplasmic oxidation potential suppresses *rdar* morhotype development in *S*. Typhimurium. However, we also noted that reducing stress, in the form of sublethal concentrations of DTT, affected the *rdar* morphotype of the *dsb* mutants. While initially revealing an enhanced *rdar* morphotype upon DTT stress, the biofilm of the mutants soon dispersed, and eventually converted to a highly mucoid morphotype.

The observed mucoid phenotype imply increased presence of an extracellular organic capsular-like polymer. A possible candidate would be cellulose already comprised in the biofilm. Yet, that genetically inactivating the cellulose-synthesis activator gene *csgD* in *dsb* mutant backgrounds rather increased the mucoid phenotype that formally speaks against this option. At the other hand we noted that *ΔdsbA-csgD* and *ΔdsbB-csgD* double mutants, while not revealing a *rdar* morphotype, still did bind the dye Congo red implicative of cellulose expression. Thus the *ΔdsbA/B-csgD* double mutants could either express structures apart from curli or cellulose ploymers binding the dye, or having alternative CsgD/AdrA independent activation of cellulose synthesis. Indeed, it has been reported that cellulose production can be activated independently of the CsgD/AdrA regulatory pathway in a mutant of c-di-GMP specific phosphodiesterase YjcC or under alternative growth conditions [57].

Apart from cellulose, many organisms contain extracellular DNA (eDNA) as an essential component of their biofilm-matrix [65]–[69]. The release of eDNA has been proposed by three mechanisms; membrane vesicles packed with DNA [70], by lysis of a fraction of bacterial population [66] and by the secretion through secretory machineries [13]. However, when exposing the mucoid colonies to deoxyribonucleases, we did not record any reducing effect in sliminess (data not shown). As *S*. Typhimurium does not express capsular Vi antigen nor poly-N-acetyl-glucosamine, respectively expressed by *S*. Typhi or *E. coli*, our observations would leave us with the slimy morphotype being derived from over-expression of colanic acid. A forthcoming approach will be to solve whether this would be the case, in order to solve whether in fact periplasmic oxidoreductase activity connect to colonic acid synthesis via CsgD.

The fact that DsbA is a defined periplasmic protein raises the question on how it affects expression of either *csgD* or the CsgD protein located in the cytoplasm. A plausible explanation comes from a model provided by Prigent-Combaret and co-workers [71]. In this model, the CpxA/CpxR response-regulator becomes activated upon accumulation of miss-folded cell envelope proteins. Such a situation could well manifest in the absence of periplasmic disulphide bond-formation, such as in the absence of DsbA. As a consequence, CpxR would become phosphorylated and start inhibiting expression of *csgD* as well as *csgA*.

The PhoQ/PhoP response regulator pair responds to a number of factors, including DsbA [72]. PhoQ/PhoP in turn regulates expression of RstA/RstB, another response regulator pathway affecting CsgD expression [73]. Thus, DsbA could reach CsgD expression through RstA/RstB by affecting PhoQ/PhoP. If so, divergent activities of the CpxA/CpxR, RstA/RstB and PhoQ/PhoP systems could explain the different contributions of *dsbA* and *dsbB* in regulating biofilm-formation under different growth conditions.

DsbA is reported to directly affect another phosphorelay system, the RcsCDB system that responds to altered disulphide bond formation [74]. In this, the response regulator gene *rcsB* appears to suppress *csgD* expression [75]. However, we could not define the accompanying increase in *csgD* transcription in our *dsb* mutants. Rather, the increase in CsgD levels in our *dsb* mutants appeared to act at a post-transcriptional stage. Hence, the models listed above may not be able to directly explain the increased CsgD levels noted for the *dsb* mutants.

Parts of the cascades initiated by response regulators are relayed to CsgD through GGDEF/EAL domain proteins. Thus, we genetically probed for the potential role of c-di-GMP and GGDEF/EAL domain proteins in the enhanced *rdar* morphotype formation exhibited by the *dsb* mutants. In these experiments, we noted that depletion of the cellular c-di-GMP lead to reappearance of mutant phenotype.

In *E. coli*, the *yddV-dos* genes code for an EAL-domain/sensor-protein complex that activate *csgBAC* expression with a heme-based redox-sensing ability [59]. Conceptually, such a complex could well fit to bridge CsgD with periplasmic redox activity through c-di-GMP signaling. However, *S*. Typhimurium lacks the *yddV-dos* genes [40]. We noted an enhanced *rdar* morphotype development in Δ*dsbA-yciR* and Δ*dsbB-yciR* double mutants. In parallel, the enhanced *rdar* morphotype development was retained in either *dsb* mutant background whether depleted for *yegE* (*STM2123*), *yfiN* (*STM2672*), *STM4551* and *yedQ* (*STM1987*), all needed for *rdar* morphotype development in the wild type background. The only GGDEF/EAL domain protein genes that appeared necessary for the *dsb* mutant-associated enhanced *rdar* morphotype development were *ydiV* (*STM1344*) and *yhjK* (*STM3615*); with *ydiV* or *yhjK* deleted, the *rdar* morphotype development was more significantly down-regulated in the Δ*dsbA/B* mutants than in the wild type strain.

However, interestingly, of note, YdiV is an EAL-domain-like protein that does not bind c-di-GMP, and elevates CsgD protein expression through repression of the activity of the master regulator of the flagella operon [76]. Through this, YdiV counteracts the inhibitory effect of EAL-domain proteins YciR and YhjH on CsgD expression. YhjK in turn has not previously been associated with *rdar* morphotype development in *S*. Typhimurium. However, a *yhjK* mutant was found severely attenuated in gut colonization in the streptomycin-treated mouse model [77].

**Figure 6:**
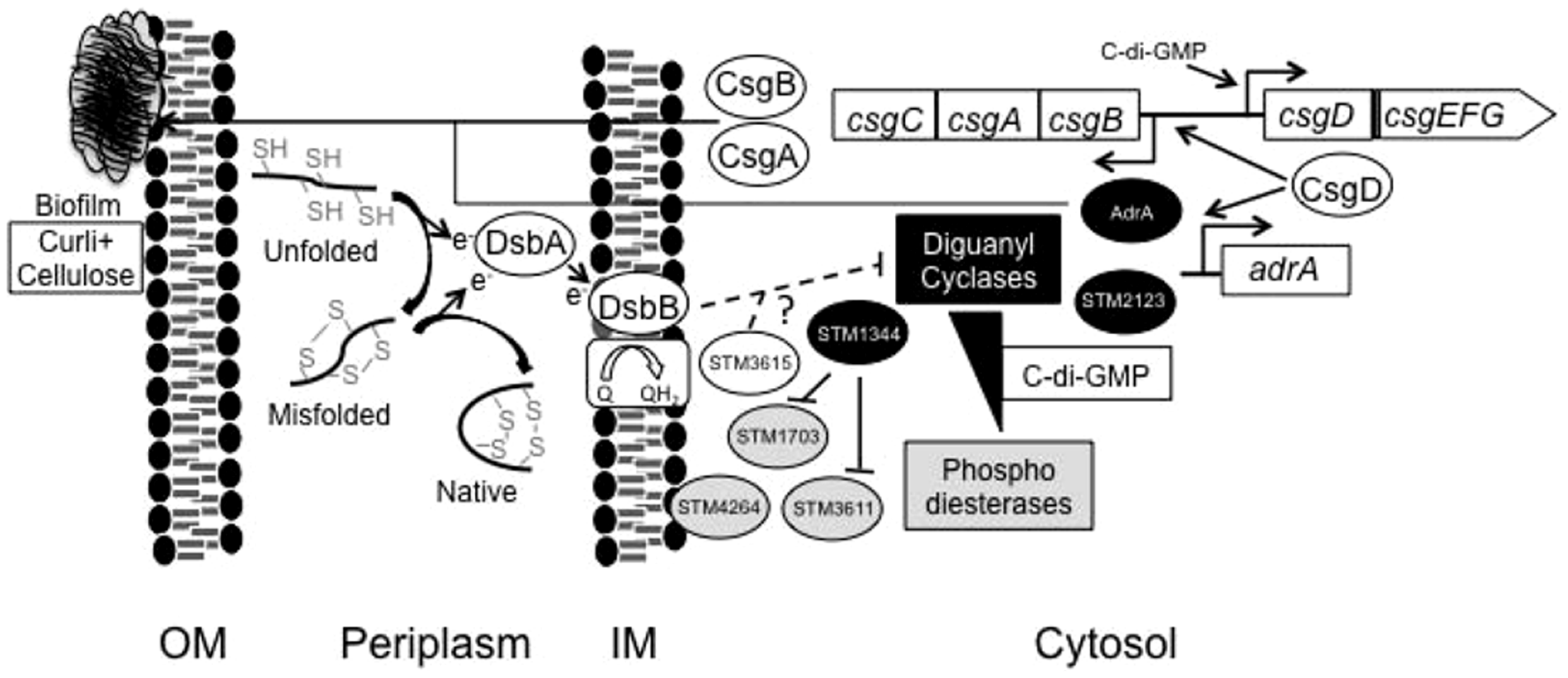
DsbA/B regulatory pathway leading to *rdar* morphotype formation in *S.* Typhimurium. In the illustration, the OM indicates outer cell membrane and IM refers inner cell membrane. The diguanyl cyclases are marked as black and the phosphodiesterases are marked as grey.

To summarize, we have observed that biofilm-formation is redox responsive. Oxidizing preferentially periplasmic proteins with sub-lethal concentrations of CuCl_2_ reduced *rdar* morphotype development. In contrast, genetically decreasing the periplasmic protein disulphide bond formation through *dsbA* or *dsbB* mutations enhanced the *rdar* morphotype development. This altered *dsb*-associated morphotype correlated with an increased CsgD and CsgA expression but relied on the GGDEF-EAL domain protein STM3615. Hence, the functional hierarchy of GGDEF/EAL domain proteins in *S*. Typhimurium could relate to redox despite a lack of an YddV-Dos system (Fig. 6). Such a parallel pathway would rely on periplasmic protein disulphide bond formation, in turn being dependent on intact DsbA/DsbB redox shuffling. As this system becomes disturbed, also the activity of selected GGDEF/EAL domain proteins, such as YdiV and YhjK, becomes altered leading to an altered CsgD activity.

## Materials and Methods

### Bacterial strains, plasmids and growth environment

The wild type *S.* Typhimurium 14028s strain (ATCC, Manassas, VA, USA) was used as reference strain in this study. All the strains and plasmids generated and used are listed in Table 2. For *trans*-complementation, the coding regions of *dsbA* and *dsbB* were cloned in pBAD30 under *ara* promoter with ribosome-binding sites in front of the start codon by following the protocols as described earlier [78]. The primers used for cloning are listed in Table S1.

**Table 2:** Strains and Plasmids.

Strains	Genotype/Property	Reference
MC5	Wild Type	ATCC 14028
NA266	14028(*ΔdsbA*)	This study
NA264	14028(*ΔdsbB*)	This study
Fia-569	14028(*ΔdsbC*)	[41]
NA-299	14028(*ΔdsbD*)	This study
NA-268	14028(*ΔdsbL*)	This study
NA-270	14028(*ΔdsbI*)	This study
Fia-410	14028(*ΔtrxA*)	[41]
Fia-412	14028(*ΔtrxB*)	[41]
Fia-406	14028(*ΔgshA*)	[41]
Fia-422	14028(*ΔgrxA*)	[41]
**Plasmids**		
VC (pBAD30)	pBAD series vector control, Amp^r^	[86]
p*dsbA* (pNA12)	The *ΔdsbA* (*STM3997*) ORF cloned in pBAD30, Amp^r^	This study
p*dsbB* (pNA13)	The *ΔdsbB* (*STM1807*) ORF cloned in pBAD30, Amp^r^	This study
pUGE13	pQF50 containing fragment +441/−685 of P*csgD*1	[55]
p*yhjH* (pRGS1)	The *yhjH* (*stm4264*) ORF cloned in pBAD30, Amp^r^	[23]

For strain propagation and cloning, bacteria were grown in ordinary Luria medium. To generate biofilm, cultures were prepared in Luria broth (LB) or on Luria agar (LA) plates without salt and grown at 28°C. When necessary, growth media were supplemented with ampicillin (100 μg/ml), chloramphenicol (10 μg/ml), kanamycin sulfate (50 μg/ml) or tetracycline (10 μg/ml). To activate the arabinose promoter in pBAD30, cultures were supplemented with L-arabinose to a final concentration of 0.1% (w/v). All antibiotics and L-arabinose were purchased from Sigma-Aldrich (St. Louis, MO, USA) unless mentioned specifically.

### Construction of mutants

Deletion mutations were carried out by one-step gene inactivation method as described earlier [79]. Mutants were selected on LA plates with antibiotic and confirmed by PCR with primers designed up- and downstream of the ORFs. The primers for mutation generation and confirmation are listed in Table S2 and Table S3 respectively. The mutations were transferred to clean 14028s *S.* Typhimurium background by P22 *int* transduction [78] to rule out the possibility of secondary mutations' effects. Deletion mutants were cured from their antibiotic resistance insert by using pCP20 plasmid as previously described [79]. The GGDEF/EAL and CsgD single (in wild type background) and double mutants (in Δ*dsbA/B* background) were created by P22 *int* phage transduction from the strains previously described [14], [56], [57], [80]. The *S.* Typhimurium virulence plasmid pSLT was cured from SR11 background to generate pSLT deficient *S.* Typhimurium [81].

### Biofilm-formation on solid and in liquid media

The bacteria were grown on LA plates overnight at 37°C and were re-suspended in PBS. The OD_600_ were adjusted to 1.0 and 5 µl was spot inoculated on LA without salt plates supplemented with Congo red (40 µg/ml) and brilliant blue (20 µg/ml) for analyzing *rdar* morphotype development. The plates were supplemented with 5 mM dithiothritol (DTT) and 1 mM, 2 mM or 3 mM CuCl_2_ where stated. In addition sterile filter papers soaked with 10 μlof 1M CuCl_2_ were applied on the plate to mimic oxidative stress. The growth was followed and pictures were taken at various time intervals post incubation.

Biofilm-formation at the air-liquid interface (pellicle formation) was carried out as described previously with slight modifications [82], [83]. Briefly, 2 ml of LB without salt in 16 ml polystyrene tubes was inoculated with 10 µl of aforesaid bacterial suspension. The tubes were left undisturbed at 28°C and followed over time for biofilm-development and adherence to the tubes' wall. Thereafter, the liquid phase was discarded by inverting the tubes carefully. The tubes were air dried and heat fixed at 60°C in hot-air oven for 1 hour. Subsequently, 300 µl of 100% methanol was added to each tube and left at room temperature (RT  = 20°C) for 15 min, with intermittent rotation at 5 min intervals to cover all the contents of adhered biofilm-mass with methanol. The methanol was replaced with 300 µl of crystal violet (1% solution in 50% methanol) and left for 10 minutes at RT with the rotations as described earlier. Finally, the stained contents were rinsed thoroughly with tap water by submerging tubes in a draining tank. The tubes were air dried by inversion and photographed. For quantification of the adherent biofilm, the bound crystal violet was dissolved in 500 µl of 30% acetic acid and OD_570_ were noted.

### Curli fimbriae expression analysis

The main subunit of curli fimbriae, the CsgA protein, was detected by the formic acid enrichment technique [84] with slight modifications. Briefly, overnight LA without salt plate culture grown at 28°C was weighed to 3 mg of scrapped bacterial colonies and washed with PBS. The washed pellet was re-suspended in TE buffer (10 mM Tris, 1 mM EDTA and 2% SDS; pH = 7.5) and boiled for 45 min at 95°C. The suspension was centrifuged at 14000 rpm and pellet was washed with dH_2_O.

After second centrifuged the pellet was re-suspended in H_2_O, dried by Speed Vac for 1 hour and re-suspended in 100% formic acid. The suspension was boiled for 15–20 min in heat block at 95°C and samples were dried again in Speed Vac for 1 hour. The denatured pellet was dissolved in 20 µl of SDS reducing sample buffer, boiled for 15 min at 95°C and loaded on 15% SDS polyacrylamide gel. The band corresponding to CsgA was visualized by Coomassie staining of the SDS-PAGE gel. Relative protein contents were compared with the one obtained from the wild type by using Image Lab (Beta 2) version 3.0.11 (Bio-Rad Laboratories).

### CsgD expression analysis

The expression of master regulator CsgD was analyzed by immunoblotting as described earlier [19]. Summarizing, overnight LA plate without salt cultures were harvested up to 5 mg (wet weight) and re-suspended in 200 µl of reducing SDS sample buffer. The samples were heated for 10–15 min at 95°C and ran on 12% SDS polyacrylamide gels. After checking the total protein contents on the Coomassie stained gel, the proteins were transferred to PVDF membrane (Amersham Hybond-P, GE Healthcare).

Detection CsgD was carried out by using a primary polyclonal peptide rabbit anti-CsgD antibody (1∶5000) and a secondary HRP-conjugated goat anti-rabbit IgG antibody (1∶10000). The chemiluminescent was detected by using ECL substrate (SuperSignal West Pico Chemiluminescent Substrate, Thermo Scientific) and Bio-Rad Gel doc machine. The protein content was analyzed as described above.

### Promotor-fusions and β-galactosidase measurements

The *csgD* promoter activity was determined from plasmid pUGE13 which contains a regulated *csgD* promoter (+441/−684 relative to the transcriptional start site) fused with *lacZ* [55]. Concomitant β-galactosidase activity was determined as described previously [85] from 7.5 and 18 hours LB without salt cultures and from 24, 48 and 72 hours LA without salt plate cultures and expressed as miller units.

### Statistical analyses

All the experiments were repeated at least three times. The graphical presentation and analysis of data were done by using the PRISM (version 5.0) software. The data for Figs. 1D, 2C, 2D, 5D and 5E were analyzed by two sided independent sample t-test and mean values with standard errors of mean (SEM) are presented.

## Supporting Information

Figure S1 **Biofilm-formation for different members of oxidoreductase systems.** The biofilm-formation was assessed on LA without salt plates supplemented with Congo red grown at 28°C. The pictures were taken 48 hours post inoculation.
(TIF)

Figure S2 **Effect of** ***ydiV-yhjK*** **gene mutations on** ***dsb*** **associated biofilm-formation.** The development of *rdar* morphotype in wild type, single, double and triple mutants on Congo red plates at 28°C. The picture was taken 48 hours post inoculation.
(TIF)

Figure S3 **Effect of oxidative stress on biofilm.** **A**) CuCl_2_ induced oxidative stress generates a dose dependent suppression of biofilm-formation irrespective of genetic background of the strain. **B**) Effect of 1M CuCl_2_ (soaked in sterile filter disc) on the rdar morphotypes of wildtype (WT) and *dsb* mutants grown on Congo red plate for 48 hours at 28°C.
(TIF)

Table S1 **Primers for cloning of** ***dsb*** **genes.**
(DOC)

Table S2 **Primers for mutagenesis.**
(DOC)

Table S3 **Primers for PCR verification of mutants.**
(DOC)

## References

[1] Gast RK, Guraya R, Guard J (2013) Salmonella enteritidis deposition in eggs after experimental infection of laying hens with different oral doses. J Food Prot. 76: 108–113. 10.4315/0362-028X.JFP-12-268 23317864

[2] Jansen A, Lahuerta-Marin A, Palm D, Purnat T, Santos CV, et al.. (2011) Annual epidemiological report 2011. European Centre for Disease Prevention and Control.

[3] Hansen-Wester I, Hensel M (2001) Salmonella pathogenicity islands encoding type III secretion systems. Microbes Infect. 3: 549–559. 10.1016/s1286-4579(01)01411-3 11418329

[4] Carter PB, Collins FM (1974) The route of enteric infection in normal mice. J Exp Med 139: 1189–1203. 4596512 10.1084/jem.139.5.1189 PMC2139651

[5] Pang T, Bhutta ZA, Finlay BB, Altwegg M (1995) Typhoid fever and other salmonellosis: a continuing challenge. Trends Microbiol. 3: 253–255. 10.1016/s0966-842x(00)88937-4 7551636

[6] Pang T, Levine MM, Ivanoff B, Wain J, Finlay BB (1998) Typhoid fever – important issues still remain. Trends Microbiol. 6: 131–133. 10.1016/s0966-842x(98)01236-0 9587187

[7] Buchwald DS, Blaser MJ (1984) A review of human salmonellosis: II. Duration of excretion following infection with nontyphi Salmonella. Rev Infect Dis. 6: 345–356. 10.1093/clinids/6.3.345 6377442

[8] Edelman R, Levine MM (1986) Summary of an international workshop on typhoid fever. Rev Infect Dis. 8: 329–349. 10.1093/clinids/8.3.329 3726393

[9] Kolker S, Itsekzon T, Yinnon AM, Lachish T (2012) Osteomyelitis due to Salmonella enterica subsp. arizonae: the price of exotic pets. Clin Microbiol Infect. 18: 167–70. 10.1111/j.1469-0691.2011.03533.x 21745257

[10] Ruby T, McLaughlin L, Gopinath S, Monack D (2012) Salmonella's long-term relationship with its host. FEMS Microbiol Rev. 36: 600–615. 10.1111/j.1574-6976.2012.00332.x 22335190

[11] Crawford RW, Reeve KE, Gunn JS (2010) Flagellated but not hyperfimbriated Salmonella enterica serovar Typhimurium attaches to and forms biofilms on cholesterol-coated surfaces. J Bacteriol. 192: 2981–2990. 10.1128/JB.01620-09 PMC2901699 20118264

[12] Römling U (2005) Characterization of the rdar morphotype, a multicellular behaviour in Enterobacteriaceae. Cell Mol Life Sci. 62: 1234–1246. 10.1007/s00018-005-4557-x PMC11139082 15818467

[13] Zogaj X, Bokranz W, Nimtz M, Römling U (2003) Production of cellulose and curli fimbriae by members of the family Enterobacteriaceae isolated from the human gastrointestinal tract. Infect Immun. 71: 4151–4158. 10.1128/IAI.71.7.4151-4158.2003 PMC162016 12819107

[14] Römling U, Rohde M, Olsen A, Normark S, Reinkoster J (2000) AgfD, the checkpoint of multicellular and aggregative behaviour in Salmonella typhimurium regulates at least two independent pathways. Molecular Microbiology 36: 10–23. 10760159 10.1046/j.1365-2958.2000.01822.x

[15] Zakikhany K, Harrington CR, Nimtz M, Hinton JCD, Römling U (2010) Unphosphorylated CsgD controls biofilm formation in Salmonella enterica serovar Typhimurium. Molecular Microbiology 77: 771–786. 20545866 10.1111/j.1365-2958.2010.07247.x

[16] Jenal U (2004) Cyclic di-guanosine-monophosphate comes of age: a novel secondary messenger involved in modulating cell surface structures in bacteria? Curr Opin Microbiol 7: 185–191. 15063857 10.1016/j.mib.2004.02.007

[17] Römling U, Gomelsky M, Galperin MY (2005) C-di-GMP: the dawning of a novel bacterial signalling system. Molecular Microbiology 57: 629–639. 16045609 10.1111/j.1365-2958.2005.04697.x

[18] Römling U, Galperin MY, Gomelsky M (2013) Cyclic di-GMP: the first 25 years of a universal bacterial second messenger. Microbiol Mol Biol Rev 77: 1–52. 23471616 10.1128/MMBR.00043-12 PMC3591986

[19] Römling U, Sierralta WD, Eriksson K, Normark S (1998) Multicellular and aggregative behaviour of Salmonella typhimurium strains is controlled by mutations in the agfD promoter. Molecular Microbiology 28: 249–264. 9622351 10.1046/j.1365-2958.1998.00791.x

[20] Ryan RP, Fouhy Y, Lucey JF, Crossman LC, Spiro S, et al (2006) Cell-cell signaling in Xanthomonas campestris involves an HD-GYP domain protein that functions in cyclic di-GMP turnover. P Natl Acad Sci USA 103: 6712–6717. 10.1073/pnas.0600345103 PMC1458946 16611728

[21] Krasteva PV, Fong JC, Shikuma NJ, Beyhan S, Navarro MV, et al (2010) Vibrio cholerae VpsT regulates matrix production and motility by directly sensing cyclic di-GMP. Science 327: 866–868. 20150502 10.1126/science.1181185 PMC2828054

[22] Ausmees N, Mayer R, Weinhouse H, Volman G, Amikam D, et al (2001) Genetic data indicate that proteins containing the GGDEF domain possess diguanylate cyclase activity. FEMS Microbiology Letters 204: 163–167. 11682196 10.1111/j.1574-6968.2001.tb10880.x

[23] Simm R, Morr M, Kader A, Nimtz M, Römling U (2004) GGDEF and EAL domains inversely regulate cyclic di-GMP levels and transition from sessility to motility. Molecular Microbiology 53: 1123–1134. 15306016 10.1111/j.1365-2958.2004.04206.x

[24] Danhorn T, Fuqua C (2007) Biofilm formation by plant-associated bacteria. Annu Rev Microbiol 61: 401–422. 17506679 10.1146/annurev.micro.61.080706.093316

[25] Elkins JG, Hassett DJ, Stewart PS, Schweizer HP, McDermott TR (1999) Protective role of catalase in Pseudomonas aeruginosa biofilm resistance to hydrogen peroxide. Appl Environ Microb 65: 4594–4600. 10.1128/aem.65.10.4594-4600.1999 PMC91612 10508094

[26] Beenken KE, Dunman PM, McAleese F, Macapagal D, Murphy E, et al (2004) Global gene expression in Staphylococcus aureus biofilms. Journal of Bacteriology 186: 4665–4684. 15231800 10.1128/JB.186.14.4665-4684.2004 PMC438561

[27] Resch A, Rosenstein R, Nerz C, Gotz F (2005) Differential gene expression profiling of Staphylococcus aureus cultivated under biofilm and planktonic conditions. Appl Environ Microb 71: 2663–2676. 10.1128/AEM.71.5.2663-2676.2005 PMC1087559 15870358

[28] Wang SY, Phillippy AM, Deng KP, Rui XQ, ZX L, et al (2010) Transcriptomic Responses of Salmonella enterica Serovars Enteritidis and Typhimurium to Chlorine-Based Oxidative Stress. Appl Environ Microb 76: 5013–5024. 10.1128/AEM.00823-10 PMC2916494 20562293

[29] Mastroeni P, Vazquez-Torres A, Fang FC, Xu Y, Khan S, et al (2000) Antimicrobial actions of the NADPH phagocyte oxidase and inducible nitric oxide synthase in experimental salmonellosis. II. Effects on microbial proliferation and host survival in vivo. J Exp Med 192: 237–248. 10899910 10.1084/jem.192.2.237 PMC2193252

[30] Hebrard M, Viala JP, Meresse S, Barras F, Aussel L (2009) Redundant hydrogen peroxide scavengers contribute to Salmonella virulence and oxidative stress resistance. J Bacteriol 191: 4605–4614. 19447905 10.1128/JB.00144-09 PMC2704729

[31] Horst SA, Jaeger T, Denkel LA, Rouf SF, Rhen M, et al (2010) Thiol peroxidase protects Salmonella enterica from hydrogen peroxide stress in vitro and facilitates intracellular growth. J Bacteriol 192: 2929–2932. 20304995 10.1128/JB.01652-09 PMC2876503

[32] Aussel L, Zhao W, Hebrard M, Guilhon AA, Viala JP, et al (2011) Salmonella detoxifying enzymes are sufficient to cope with the host oxidative burst. Mol Microbiol 80: 628–640. 21362067 10.1111/j.1365-2958.2011.07611.x

[33] Denkel LA, Horst SA, Rouf SF, Kitowski V, Bohm OM, et al (2011) Methionine sulfoxide reductases are essential for virulence of Salmonella typhimurium. PLoS One 6: e26974. 22073230 10.1371/journal.pone.0026974 PMC3206869

[34] Barraud N, Hassett DJ, Hwang SH, Rice SA, Kjelleberg S, et al (2006) Involvement of nitric oxide in biofilm dispersal of Pseudomonas aeruginosa. J Bacteriol. 188: 7344–7353. 10.1128/JB.00779-06 PMC1636254 17050922

[35] Plate L, Marletta MA (2012) Nitric oxide modulates bacterial biofilm formation through a multicomponent cyclic-di-GMP signaling network. Mol Cell. 46: 449–460. 10.1016/j.molcel.2012.03.023 PMC3361614 22542454

[36] Berndt C, Lillig CH, Holmgren A (2008) Thioredoxins and glutaredoxins as facilitators of protein folding. Bba-Mol Cell Res 1783: 641–650. 10.1016/j.bbamcr.2008.02.003 18331844

[37] Inaba K (2009) Disulfide Bond Formation System in Escherichia coli. J Biochem 146: 591–597. 19567379 10.1093/jb/mvp102

[38] Holmgren A, Lu J (2010) Thioredoxin and thioredoxin reductase: Current research with special reference to human disease. Biochem Bioph Res Co 396: 120–124. 10.1016/j.bbrc.2010.03.083 20494123

[39] Lin D, Kim B, Slauch JM (2009) DsbL and DsbI contribute to periplasmic disulfide bond formation in Salmonella enterica serovar Typhimurium. Microbiology. 155: 4014–4024. 10.1099/mic.0.032904-0 PMC2889420 19797361

[40] McClelland M, Sanderson KE, Spieth J, Clifton SW, Latreille P (2001) Complete genome sequence of Salmonella enterica serovar Typhimurium LT2. Nature 413: 852–856. 11677609 10.1038/35101614

[41] Bjur E, Eriksson-Ygberg S, Aslund F, Rhen M (2006) Thioredoxin 1 promotes intracellular replication and virulence of Salmonella enterica serovar Typhimurium. infection and Immunity 74: 5140–5151. 16926406 10.1128/IAI.00449-06 PMC1594827

[42] Lin D, Rao CV, Slauch JM (2008) The Salmonella SPI1 type three secretion system responds to periplasmic disulfide bond status via the flagellar apparatus and the RcsCDB system. J Bacteriol 190: 87–97. 17951383 10.1128/JB.01323-07 PMC2223759

[43] Negrea A, Bjur E, Puiac S, Ygberg SE, Aslund F, et al (2009) Thioredoxin 1 Participates in the Activity of the Salmonella enterica Serovar Typhimurium Pathogenicity Island 2 Type III Secretion System. Journal of Bacteriology 191: 6918–6927. 19767428 10.1128/JB.00532-09 PMC2772483

[44] Kim YH, Lee Y, Kim S, Yeom J, Yeom S, et al (2006) The role of periplasmic antioxidant enzymes (superoxide dismutase and thiol peroxidase) of the Shiga toxin-producing Escherichia coli O157: H7 in the formation of biofilms. Proteomics 6: 6181–6193. 17133368 10.1002/pmic.200600320

[45] Lee Y, Kim Y, Yeom S, Kim S, Park S, et al (2008) The role of disulfide bond isomerase A (DsbA) of Escherichia coli O157: H7 in biofilm formation and virulence. FEMS Microbiology Letters 278: 213–222. 18067575 10.1111/j.1574-6968.2007.00993.x

[46] Lee Y, Oh S, Park W (2009) Inactivation of the Pseudomonas putida KT2440 dsbA gene promotes extracellular matrix production and biofilm formation. FEMS Microbiology Letters 297: 38–48. 19500143 10.1111/j.1574-6968.2009.01650.x

[47] Bijtenhoorn P, Mayerhofer H, Muller-Dieckmann J, Utpatel C, Schipper C, et al (2011) A Novel Metagenomic Short-Chain Dehydrogenase/Reductase Attenuates Pseudomonas aeruginosa Biofilm Formation and Virulence on Caenorhabditis elegans. PLoS One 6: e26278. 22046268 10.1371/journal.pone.0026278 PMC3202535

[48] Suo YJ, Huang YY, Liu YH, Shi CL, Shi XM (2012) The Expression of Superoxide Dismutase (SOD) and a Putative ABC Transporter Permease Is Inversely Correlated during Biofilm Formation in Listeria monocytogenes 4b G. PLoS One. 7: e48467. 10.1371/journal.pone.0048467 PMC3485238 23119031

[49] Achard ME, Stafford SL, Bokil NJ, Chartres J, Bernhardt PV, et al (2012) Copper redistribution in murine macrophages in response to Salmonella infection. Biochem J 444: 51–57. 22369063 10.1042/BJ20112180

[50] Anwar N, Sem XH, Rhen M (2013) Oxidoreductases that Act as Conditional Virulence Suppressors in Salmonella enterica Serovar Typhimurium. PLoS One 8: e64948. 23750221 10.1371/journal.pone.0064948 PMC3672137

[51] Römling U, Rohde M (1999) Flagella modulate the multicellular behavior of Salmonella typhimurium on the community level. FEMS Microbiol Lett 180: 91–102. 10547449 10.1111/j.1574-6968.1999.tb08782.x

[52] Hammar M, Arnqvist A, Bian Z, Olsen A, Normark S (1995) Expression of two csg operons is required for production of fibronectin- and Congo red-binding curli polymers in Escherichia coli K-12. Molecular Microbiology 18: 661–670. 10.1111/j.1365-2958.1995.mmi_18040661.x. 8817489

[53] Collinson SK, Doig PC, Doran JL, Clouthier S, Trust TJ, et al (1993) Thin, aggregative fimbriae mediate binding of Salmonella enteritidis to fibronectin. J Bacteriol. 175: 12–18. 10.1128/jb.175.1.12-18.1993 PMC196092 8093237

[54] Gerstel U, Römling U (2003) The csgD promoter, a control unit for biofilm formation in Salmonella typhimurium. Res Microbiol 154: 659–667. 14643403 10.1016/j.resmic.2003.08.005

[55] Gerstel U, Kolb A, Römling U (2006) Regulatory components at the csgD promoter – additional roles for OmpR and integration host factor and role of the 5′ untranslated region. FEMS Microbiol Lett 261: 109–117. 16842367 10.1111/j.1574-6968.2006.00332.x

[56] Kader A, Simm R, Gerstel U, Morr M, Römling U (2006) Hierarchical involvement of various GGDEF domain proteins in rdar morphotype development of Salmonella enterica serovar Typhimurium. Molecular Microbiology 60: 602–616. 16629664 10.1111/j.1365-2958.2006.05123.x

[57] Simm R, Lusch A, Kader A, Andersson M, Römling U (2007) Role of EAL-containing proteins in multicellular behavior of Salmonella enterica serovar typhimurium. Journal of Bacteriology 189: 3613–3623. 17322315 10.1128/JB.01719-06 PMC1855888

[58] Sommerfeldt N, Possling A, Becker G, Pesavento C, Tschowri N, et al (2009) Gene expression patterns and differential input into curli fimbriae regulation of all GGDEF/EAL domain proteins in Escherichia coli. Microbiology. 155: 1318–1331. 10.1099/mic.0.024257-0 19332833

[59] Tagliabue L, Maciag A, Antoniani D, Landini P (2010) The yddV-dos operon controls biofilm formation through the regulation of genes encoding curli fibers' subunits in aerobically growing Escherichia coli. Fems Immunol Med Mic 59: 477–484. 10.1111/j.1574-695X.2010.00702.x 20553324

[60] Ahmed I (2013) Regulatory netwroks of c-di-GMP signalling involved in biofilm formation motility and host pathogen interactions in Salmonella typhimurium. PhD Thesis. Karolinska Institutet, Sweden. ISBN 978-91-7549-224-7. Pp 27–29.

[61] Costerton JW, Stewart PS, Greenberg EP (1999) Bacterial biofilms: A common cause of persistent infections. Science 284: 1318–1322. 10334980 10.1126/science.284.5418.1318

[62] Bardwell JC, Lee JO, Jander G, Martin N, Belin D, et al (1993) A pathway for disulfide bond formation in vivo. Proc Natl Acad Sci U S A 90: 1038–1042. 8430071 10.1073/pnas.90.3.1038 PMC45806

[63] Denoncin K, Collet JF (2012) Disulfide Bond Formation in the Bacterial Periplasm: Major Achievements and Challenges Ahead. Antioxid Redox Signal. Antioxid Redox Signal. 19: 63–71. 10.1089/ars.2012.4864 PMC3676657 22901060

[64] Hiniker A, Collet JF, Bardwell JCA (2005) Copper stress causes an in vivo requirement for the Escherichia coli disulfide isomerase DsbC. Journal of Biological Chemistry 280: 33785–33791. 16087673 10.1074/jbc.M505742200

[65] Whitchurch CB, Tolker-Nielsen T, Ragas PC, Mattick JS (2002) Extracellular DNA required for bacterial biofilm formation. Science 295: 1487. 11859186 10.1126/science.295.5559.1487

[66] Dinesh SD, Grundmann H, Pitt TL, Römling U (2003) European-wide distribution of Pseudomonas aeruginosa clone C. Clin Microbiol Infect. 9: 1228–1233. 10.1111/j.1469-0691.2003.00793.x 14686989

[67] Bockelmann U, Janke A, Kuhn R, Neu TR, Wecke J, et al (2006) Bacterial extracellular DNA forming a defined network-like structure. FEMS Microbiology Letters 262: 31–38. 16907736 10.1111/j.1574-6968.2006.00361.x

[68] Izano EA, Amarante MA, Kher WB, Kaplan JB (2008) Differential roles of poly-N-acetylglucosamine surface polysaccharide and extracellular DNA in Staphylococcus aureus and Staphylococcus epidermidis biofilms. Appl Environ Microbiol 74: 470–476. 18039822 10.1128/AEM.02073-07 PMC2223269

[69] Ma LM, Conover M, Lu HP, Parsek MR, Bayles K, et al (2009) Assembly and Development of the Pseudomonas aeruginosa Biofilm Matrix. Plos Pathogens 5: e1000354. 19325879 10.1371/journal.ppat.1000354 PMC2654510

[70] Römling U, Bokranz W, Rabsch W, Zogaj X, Nimtz M, et al (2003) Occurrence and regulation of the multicellular morphotype in Salmonella serovars important in human disease. Int J Med Microbiol 293: 273–285. 14503792 10.1078/1438-4221-00268

[71] Prigent-Combaret C, Brombacher E, Vidal O, Ambert A, Lejeune P, et al (2001) Complex regulatory network controls initial adhesion and biofilm formation in Escherichia coli via regulation of the csgD gene. J Bacteriol 183: 7213–7223. 11717281 10.1128/JB.183.24.7213-7223.2001 PMC95571

[72] Lippa AM, Goulian M (2012) Perturbation of the oxidizing environment of the periplasm stimulates the PhoQ/PhoP system in Escherichia coli. J Bacteriol 194: 1457–1463. 22267510 10.1128/JB.06055-11 PMC3294871

[73] Ogasawara H, Hasegawa A, Kanda E, Miki T, Yamamoto K (2007) Genomic SELEX search for target promoters under the control of the PhoQP-RstBA signal relay cascade. J Bacteriol 189: 4791–4799. 17468243 10.1128/JB.00319-07 PMC1913430

[74] Majdalani N, Gottesman S (2005) The Rcs phosphorelay: a complex signal transduction system. Annu Rev Microbiol. 59: 379–405. 10.1146/annurev.micro.59.050405.101230 16153174

[75] Vianney A, Jubelin G, Renault S, Dorel C, Lejeune P, et al (2005) Escherichia coli tol and rcs genes participate in the complex network affecting curli synthesis. Microbiology. 151: 2487–2497. 10.1099/mic.0.27913-0 16000739

[76] Simm R, Remminghorst U, Ahmad I, Zakikhany K, Römling U (2009) A role for the EAL-like protein STM1344 in regulation of CsgD expression and motility in Salmonella enterica serovar Typhimurium. J Bacteriol. 191: 3928–3937. 10.1128/JB.00290-09 PMC2698401 19376870

[77] Ahmad I, Lamprokostopoulou A, Guyon SL, Streck E, Barthel M, et al (2011) Complex c-di-GMP signaling networks mediate transition between virulence properties and biofilm formation in Salmonella enterica serovar Typhimurium. PLoS One. 6: e28351. 10.1371/journal.pone.0028351 PMC3229569 22164276

[78] Sambrook J, Russel DW (2001) Molecular cloning 3rd ed, vol. 3. Cold Spring Harbor Laboratory Press, New York.

[79] Datsenko KA, Wanner BL (2000) One-step inactivation of chromosomal genes in Escherichia coli K-12 using PCR products. Proc Natl Acad Sci U S A 97: 6640–6645. 10829079 10.1073/pnas.120163297 PMC18686

[80] Schmieger H (1972) Phage P22-mutants with increased or decreased transduction abilities. Mol Gen Genet 119: 75–88. 4564719 10.1007/BF00270447

[81] Tinge SA, Curtiss R 3rd (1990) Isolation of the replication and partitioning regions of the Salmonella typhimurium virulence plasmid and stabilization of heterologous replicons. J Bacteriol 172: 5266–5277. 2203747 10.1128/jb.172.9.5266-5277.1990 PMC213189

[82] Stepanovic S, Vukovic D, Dakic I, Savic B, Svabic-Vlahovic M (2000) A modified microtiter-plate test for quantification of staphylococcal biofilm formation. J Microbiol Methods 40: 175–179. 10699673 10.1016/s0167-7012(00)00122-6

[83] Stepanovic S, Cirkovic I, Ranin L, Svabic-Vlahovic M (2004) Biofilm formation by Salmonella spp. and Listeria monocytogenes on plastic surface. Lett Appl Microbiol 38: 428–432. 15059216 10.1111/j.1472-765X.2004.01513.x

[84] Römling U, Bokranz W, Gerstel U, Lunsforf H, Nimtz M, et al.. (2003) Dissection of the genetic pathway leading to multicellular behaviour in Salmonella enterica serovar Tyhpimurium and other enteribacteriacear, p. 231–259. *In* M. Wilson and D. Devine (ed.), Medical Implications of Biofilm. Cambridge University Press, Cambridge, UK.

[85] Miller JH (ed) (1972) Experiments in molecular genetics. Cold Spring Harbor Laboratory Press, U.S., New York.

[86] Guzman LM, Belin D, Carson MJ, Beckwith J (1995) Tight regulation, modulation, and high-level expression by vectors containing the arabinose PBAD promoter. J Bacteriol 177: 4121–4130. 7608087 10.1128/jb.177.14.4121-4130.1995 PMC177145

