# Supplementary figures and images for: Modulation of Biofilm-Formation in Salmonella enterica Serovar Typhimurium by the Periplasmic DsbA/DsbB Oxidoreductase System Requires the GGDEF-EAL Domain Protein STM3615
Source: PLoS One. 2014 Aug 25;9(8):e106095. doi: 10.1371/journal.pone.0106095 (PMC4143323; doi:10.1371/journal.pone.0106095)

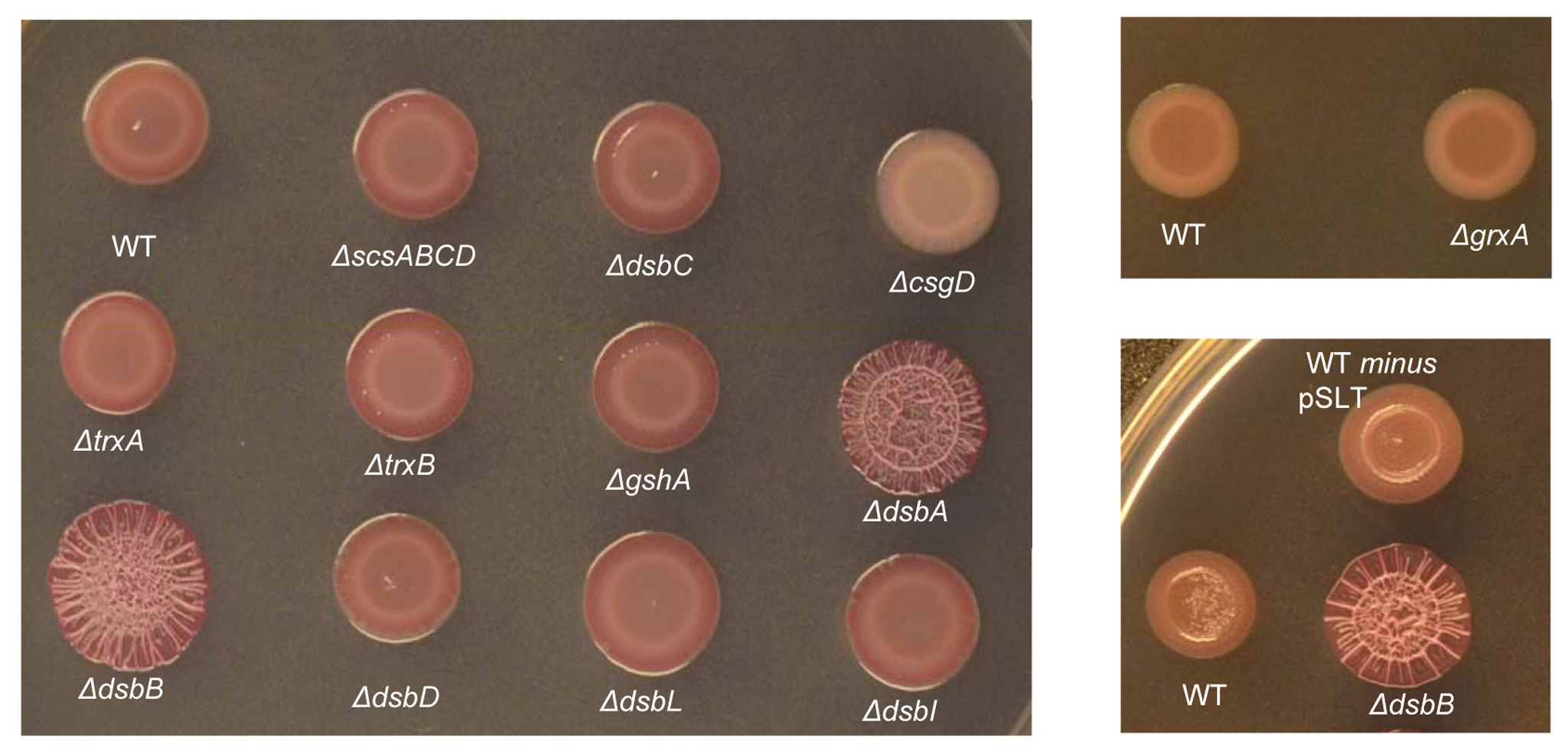

Supplement: Figure S1 — Biofilm-formation for different members of oxidoreductase systems. The biofilm-formation was assessed on LA without salt plates supplemented with Congo red grown at 28°C. The pictures were taken 48 hours post inoculation. (TIF) [file pone.0106095.s001.tif]

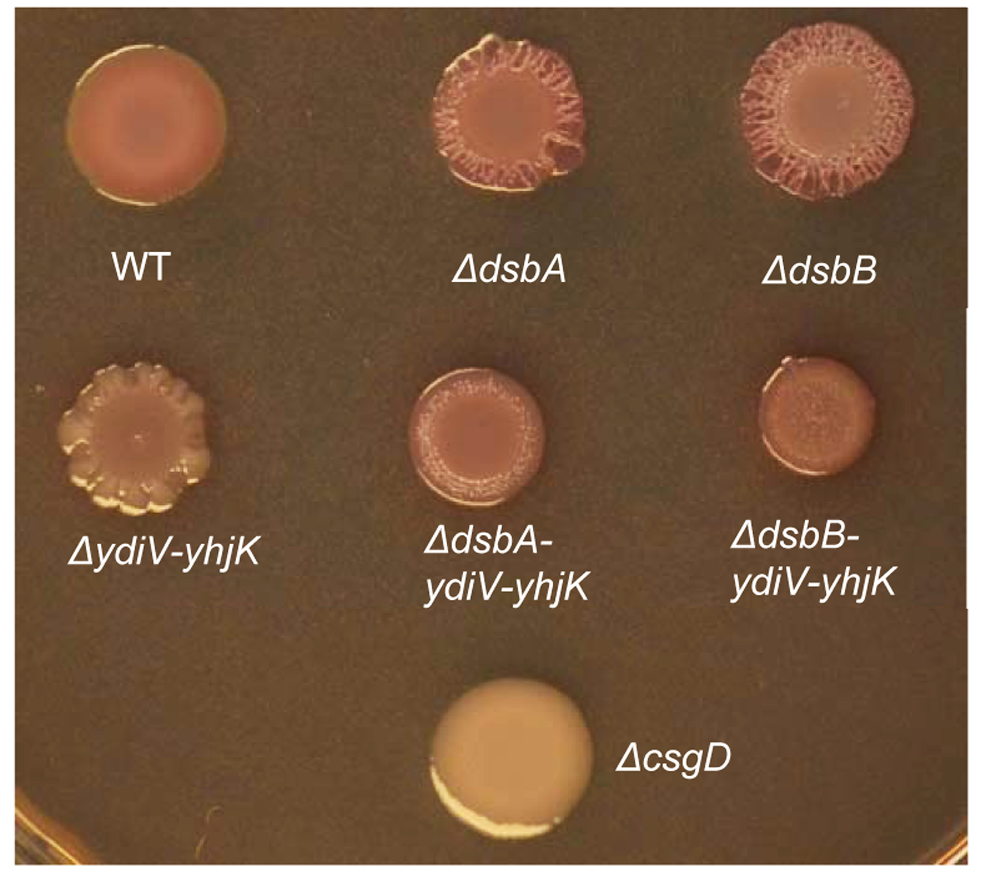

Supplement: Figure S2 — Effect of ydiV-yhjK gene mutations on dsb associated biofilm-formation. The development of rdar morphotype in wild type, single, double and triple mutants on Congo red plates at 28°C. The picture was taken 48 hours post inoculation. (TIF) [file pone.0106095.s002.tif]

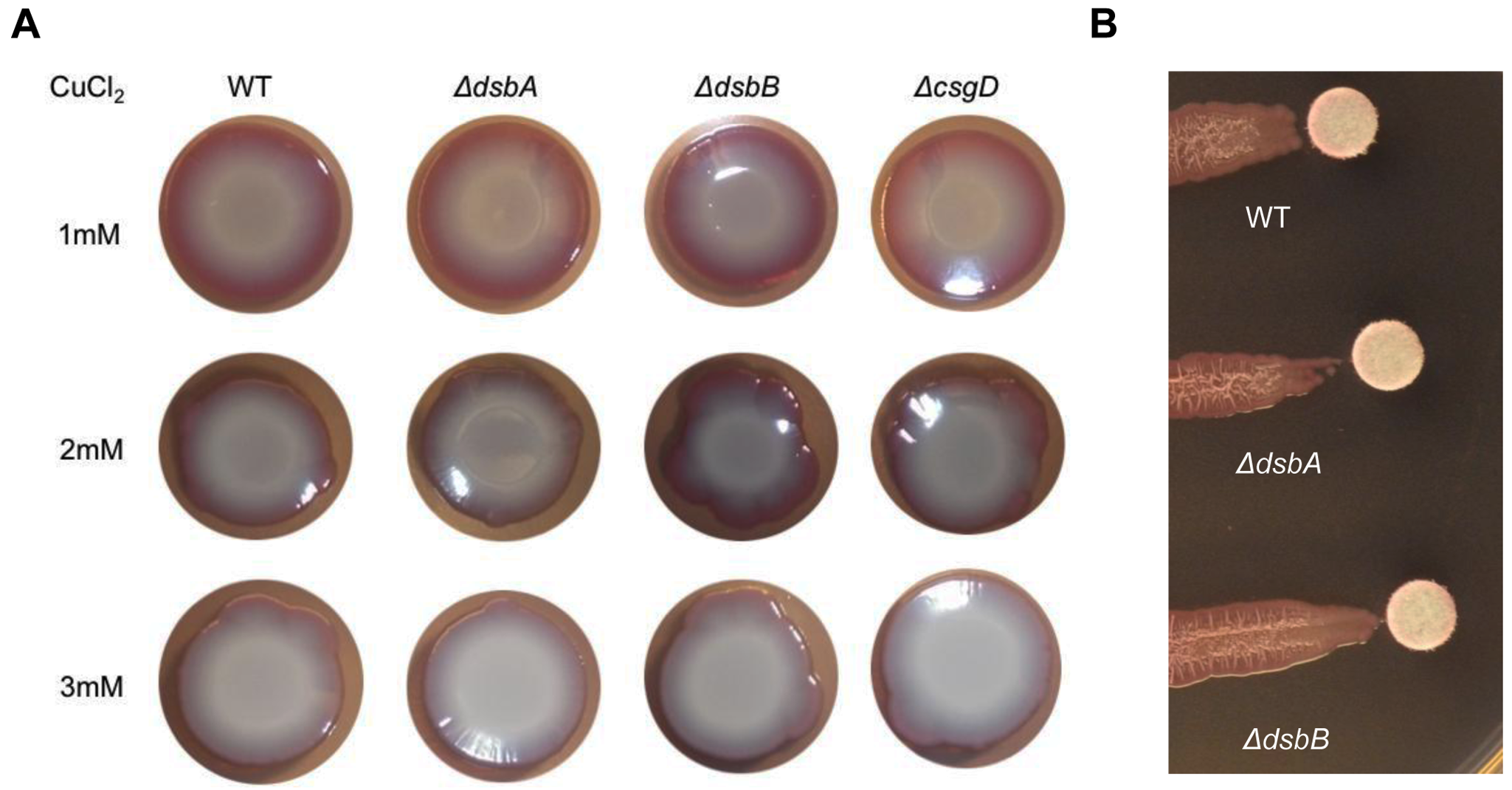

Supplement: Figure S3 — Effect of oxidative stress on biofilm. A) CuCl2 induced oxidative stress generates a dose dependent suppression of biofilm-formation irrespective of genetic background of the strain. B) Effect of 1M CuCl2 (soaked in sterile filter disc) on the rdar morphotypes of wildtype (WT) and dsb mutants grown on Congo red plate for 48 hours at 28°C. (TIF) [file pone.0106095.s003.tif]
