# Supplementary material for: Modulation of Biofilm-Formation in Salmonella enterica Serovar Typhimurium by the Periplasmic DsbA/DsbB Oxidoreductase System Requires the GGDEF-EAL Domain Protein STM3615
Source: PLoS One. 2014 Aug 25;9(8):e106095. doi: 10.1371/journal.pone.0106095 (PMC4143323; doi:10.1371/journal.pone.0106095)
Supplement: Table S1 — Primers for cloning of dsb genes. (DOC) [file pone.0106095.s004.doc]

**Table S1. Primers for cloning of *dsb*** genes

| Plasmid | Sequence *(5´ – 3´)* | Target plasmid |
| --- | --- | --- |
| pNA12 | F_w_: GACGAATTC*AGGAGGA*CGGAGAGAGTTGATCATGAA | The *ΔdsbA* gene cloned between BamH1 and XbaI site of pBAD30 |
|  | R_v_: CATCTAGAGTGACCGGCGTTCTTTTT |  |
| pNA13 | F_w_: AGACGAATTC*AGGAGGA*TACGCATGTTGCAGGGAA | The *ΔdsbB* gene cloned between BamHI and XbaI site of pBAD30 |
|  | R_v_: CATCTAGATCAACGACCGAACAGGTC |  |

Fw = Forward primer; Rv = Reverse primer.
Restriction sites are underlined and RBS is italicized.
