# Supplementary material for: Modulation of Biofilm-Formation in Salmonella enterica Serovar Typhimurium by the Periplasmic DsbA/DsbB Oxidoreductase System Requires the GGDEF-EAL Domain Protein STM3615
Source: PLoS One. 2014 Aug 25;9(8):e106095. doi: 10.1371/journal.pone.0106095 (PMC4143323; doi:10.1371/journal.pone.0106095)
Supplement: Table S2 — Primers for mutagenesis. (DOC) [file pone.0106095.s005.doc]

**Table S2. Primers for** mutagenesis

| **Primer** | **Sequence *(5´ – 3´)^*^*** | **Target gene** | **Reference** |
| --- | --- | --- | --- |
| F*dsbA*rec | ATGAAAAAGATTTGGCTGGCGCTGGCTGGTATGGTTTTAGGTGTAGGCTGGAGCTGCTTC | *dsbA* | *stm3997* |
| R*dsbA*rec | ACATCCATGCTGCTCGTATCCATGCCTTGTGGGTTAATCTCATATGAATATCCTCCTTAG |
| F*dsbB*rec | ATGTTGCGATTTTTAAACCAGTGCTCACGGGGTCGGGGCGGTGTAGGCTGGAGCTGCTTC | *dsbB* | *stm1807* |
| R*dsbB*rec | TCAACGACCGAACAGGTCGCGTTTTTTAGGCTTAAATGCCCATATGAATATCCTCCTTAG |
| F*dsbD*rec | ATGGCTCAACGCATCTTTACGCTGATCCTGCTGCTGTGCAGTGTAGGCTGGAGCTGCTTC | *dsbD* | *stm4323* |
| R*dsbD*rec | ACAGGCCACGCACCAGTCGGCGTAGAAATCCAGCATAACGCATATGAATATCCTCCTTAG |
| F*dsbI*rec | ATGGATTTTATTAAGGGATTATGGCGAGACCTACGCGCAAGTGTAGGCTGGAGCTGCTTC | *dsbI* | *stm3194* |
| R*dsbI*rec | TTATTTGCCTCTGGCCAGTTTCAATGCCCAGGCGCCGCTCCATATGAATATCCTCCTTAG |
| F*dsbL*rec | ATGTCATCTAAATGGATTACCTCTTTATTTAAAAGCGTAGGTGTAGGCTGGAGCTGCTTC | *dsbL* | *stm3193* |
| R*dsbL*rec | ACGGCAGGATCTTTCAGCGCCGCTTCAAAATCAGCCTGGCCATATGAATATCCTCCTTAG |

***^*^****Underlined sequences are homologous to template plasmids; pKD3 or pKD4*
