# Supplementary material for: Modulation of Biofilm-Formation in Salmonella enterica Serovar Typhimurium by the Periplasmic DsbA/DsbB Oxidoreductase System Requires the GGDEF-EAL Domain Protein STM3615
Source: PLoS One. 2014 Aug 25;9(8):e106095. doi: 10.1371/journal.pone.0106095 (PMC4143323; doi:10.1371/journal.pone.0106095)
Supplement: Table S3 — Primers for PCR verification of mutants. (DOC) [file pone.0106095.s006.doc]

**Table S3. Primers for PCR verification of mutants**

| **Primer** | **Sequence *(5´ – 3´)**** |
| --- | --- |
| F*dsbA*(c) | TACTGGCAGCGACAGACA |
| R*dsbA*(c) | TTGGCTTAATACGCTGTG |
| F*dsbB*(c) | GCACGAATTGAATTGGTT |
| R*dsbB*(c) | TCCCTGATGTGGTGATTA |
| F*dsbD*(c) | AGCAAGCGCTTATCGACT |
| R*dsbD*(c) | TCCCTTGCGTCTCCAGTA |
| F*dsbI*(c) | ACGGCAAGTACCTTATCT |
| R*dsbI*(c) | CGTTCAGTTTCAAAGAAC |
| F*dsbL*(c) | ATTGATGTGCTGTCGGAT |
| R*dsbL*(c) | ATTTTGGCAACGTCATAG |

***^*^*** *Sequences are homologous to upstream and downstream of the target genes.*
